# Clostridial spores as live 'Trojan horse' vectors for cancer gene therapy: comparison with viral delivery systems

**DOI:** 10.1186/1479-0556-6-8

**Published:** 2008-02-17

**Authors:** Ming Q Wei, Ruimei Ren, David Good, Jozef Anné

**Affiliations:** 1 Department of Medicine, University of Queensland, Prince Charles Hospital, Brisbane, Queensland, 4032, Australia; 2 Division of Molecular and Gene Therapies, Griffith Institute for Health and Medical Research, GH1, Griffith University, Gold Coast, Queensland, 4222, Australia; 3 Tumour Hospital, Shandong Academy of Medical Sciences, Jinan, Shandong Province, PR China; 4 Rega Institute for Medical Research, Minderbroedersstraat 10, B-3000 Leuven, Belgium

## Abstract

Solid tumours account for 90% of all cancers. Gene therapy represents a potential new modality for their treatment. Up to now, several approaches have been developed, but the most efficient ones are the viral vector based gene therapy systems. However, viral vectors suffer from several deficiencies: firstly most vectors currently in use require intratumoural injection to elicit an effect. This is far from ideal as many tumours are inaccessible and many may have already spread to other parts of the body, making them difficult to locate and inject gene therapy vectors into. Second, because of cell heterogeneity within a given cancer, the vectors do not efficiently enter and kill every cancer cell. Third, hypoxia, a prevalent characteristic feature of most solid tumours, reduces the ability of the viral vectors to function and decreases viral gene expression and production. Consequently, a proportion of the tumour is left unaffected, from which tumour regrowth occurs. Thus, cancer gene therapy has yet to realise its full potential.

The facultative or obligate anaerobic bacteria have been shown to selectively colonise and regerminate in solid tumours when delivered systemically. Among them, the clostridial spores were easy to produce, stable to store and safe to use as well as having extensive oncolytic ability. However, research in animals and humans has shown that oncolysis was almost always interrupted sharply at the outer rim of the viable tumour tissue where the blood supply was sufficient. These clostridial spores, though, could serve as "Trojan horse" for cancer gene therapy. Indeed, various spores harbouring genes for cancerstatic factors, prodrug enzymes, or proteins or cytokines had endowed with additional tumour-killing capability. Furthermore, combination of these "Trojan horses" with conventional chemotherapy or radiation therapies often significantly perform better, resulting in the "cure" of solid tumours in a high percentage of animals.

It is, thus, not too difficult to predict the potential outcomes for the use of clostridial spores as "Trojan horse" vectors for oncolytic therapy when compared with viral vector-mediated cancer therapy for it be replication-deficient or competent. However, to move the "Trojan horse" to a clinic, though, additional requirements need to be satisfied (i) target tumours only and not anywhere else, and (ii) be able to completely kill primary tumours as well as metastases. Current technologies are in place to achieve these goals.

## Background

Gene therapy represents a potential new modality for the treatment of cancer and it is developing with a very fast pace [1]. By the end of 2006, 854 protocols have been proposed or trailed in the clinic setting for various cancers, accounting for 66.6% of all gene therapy trials in humans [2]. This has reflected the fact that cancer has become the leading causes of death in Western world [3].

The key to a successful gene therapy is the vector system. Various vectors have been developed with unique features, including viral and non-viral based therapy systems. Although each has its own advantages and disadvantages, the replication-competent oncolytic viral vectors are the most promising amongst existing ones [4,5]. However, due to the complex nature of cancers, these vectors suffer from several deficiencies: firstly the majority of vectors currently in use requires intratumoural injection to elicit an effect. While this might be useful in some cases, it has limited applicability and, in fact, far from ideal as many tumours are inaccessible and spread to other areas of the body making them difficult to locate and treat. Second, most vectors do not have the capacity to efficiently enter and kill every tumour cell. Consequently, a proportion of the tumour mass is left unaffected, from which tumour regrowth occurs [6]. Although modest therapeutic responses have been associated with the convincing transgene expression in tumour tissues isolated from patients, unequivocal proof of clinical efficacy is yet to be achieved. It is, thus, fair to say that cancer gene therapy has yet to realise its full potential.

Of all cancer diagnosed, 90% of these are solid tumours. Recent understanding of the unique pathology of solid tumours has shed light on the disappointing nature of these new therapies and now demands rational and innovative design of vectors. All solid tumours, when they grow more than 2 mm diameter in size, undergo angiogenesis that results in biological changes and adaptive metabolisms, i.e.: formation of defective vessels, appearance of hypoxic areas, and emergence of heterogeneous tumour cell population [7]. This micro milieu provides a haven for anaerobic bacteria. The strictly anaerobic *Clostridia *have several advantages over others as clostridial spores specifically colonise and germinate into vegetative cells in the hypoxic regions of solid tumours, causing tumour lysis and destruction. Early trials in the 70's of non pathogenic strains in human had shown plausible safety. However, existing knowledge indicated that oncolysis was almost always interrupted sharply at the outer rim of viable tumour tissue, thus, combinational approaches have to be implemented, such as with radiofrequency therapy [8,9]. A new trial of a non pathogenic strain of *C. novyi *in combination with microtubule-interacting chemotherapeutic agents, including vinorelbine and docetaxel and demonstrated very promising results. A phase 1 trial of the combined approach in patients is in progress [10].

The intrinsic property of tumour-targeted colonisation of clostridia enables them to serve as "Trojan horses" for the delivery of genes for cancer therapy. Indeed, clostridial spores that were genetically manipulated to harbour genes for cancerstatic factors, prodrug converting enzymes, or cytokines to improve their innate oncolytic activity have been developed, including our work and others [11,12]. Furthermore, these vectors used in combination with conventional chemotherapy or radiation therapies often perform better [12]. The notable advantages of using clostridial spores are not only their innate ability for tumour colonisation and destruction, but also the seemingly unlimited capacity of these vectors to carry exogenous genes. This characteristic beckons for the development of novel ideas to equip clostridial spores with gene combinations that may break immune suppression or elicit a strong anti-tumour response to eliminate tumour metastases, the ultimate cause of cancer death [13].

This review briefly describes the viral vectors, including the replication defective vectors, the targeted vectors and replication-competent oncolytic vectors, and their use in cancer gene therapy as well as their advantages and disadvantages. Subsequently, we will focus on the development of clostridial spores as "Trojan horse" vectors for cancer therapy, the mechanisms involved, and the foreseeable promises and problems when compared with existing viral vector systems.

## The development and use of viral vector systems for cancer gene therapy

### (A) Retroviral vector systems

Murine Moloney leukaemia virus (MoMLV)-based retroviral vector is one of the earliest systems that were developed for gene therapy. The vector has unique ability to transduce dividing cells [14]. Tumour cells are growing fast and continuously dividing, such as in the case of glioma, providing a rational for *in vivo *use of the vector system. However, studies in animal models have shown that poor vector penetration is always a problem *in vivo*. MoMLV vector rarely travelled away from the injection sites [15]. Due to this relative inefficiency of transducing target cells, replication competent MoMLV has been developed. A recent report from Kasahara's group showed complete transduction of human U87 glioma xenografts in nude mice after a single intracranial (*i.c*.) injection of replication-competent MoMLV [16]. Viral envelope was stained positively in glioma cells away from the injection sites. Most importantly, no virus was detected in any non-tumour tissues, showing strict tumour specificity. In another study, replication competent retrovirus harbouring a herpes simplex thymidine kinase (HSV TK) gene was able to sensitize glioma cells in Lewis rats and achieved an up to 20% longer-term survives (40 days).

Overall, more than 23% of all gene therapy trials in patients for various diseases have used a replication-defective or competent MoMLV vector system. Existing studies in animal experiments have shown that the vector system was relatively safe and non-toxic. The case against retroviral vector systems is potential problems related to activation of cellular oncogenes and inactivation of tumour suppressor genes by insertional mutagenesis. This was true in the case of using MoMLV to transducer bone marrow stem cells for the therapy of severe combined immune deficiency syndrome (SCIDs), 4 out of 11 treated children have now developed leukaemia [17]. In addition to improving the safety, there are also studies that showed that the dissemination of the vectors in solid tumours needed to be improved in order to reach clinical efficacy.

Lentiviral vector is a new type, more complicated retroviral vectors, which are primarily based on Human or Bovine Immunodeficiency Virus. They have all the unique features of MoMLV and have been shown to transduce post mitotic cells *in vitro *and *in vivo *[18]. Studies with the Human Immunodeficiency Virus (HIV)-based vectors have shown efficient gene transfer in tumour models. Since HIV is a human pathogen, there was a tenancy of reluctant use in human patients even though a clinical trial assessing use of lentoviral vector for the therapy of AIDS is underway [19]. Thus, several bovine based vector systems were developed, which have the advantage of less or no pathogenicity in humans or any seroconversion to the original pathogen, thus, it was assumed that they carry less disease potential than possible seroconversion of vectors derived from human pathogens. We have also developed a bovine lentiviral vector system based on the Jembrana Disease virus (JDV) [20,21]. JDV only causes disease in a specific species of cattle in the Jembrana district in Bali, Indonesia, but does not affect humans. Pathological changes include intense non-follicular lympho-proliferation by reticulum and lymphoblastoid cells in lymphoid organs. Follow up protein and genome sequence studies have confirmed that JDV has a genome of 7732 nt and structure and organisation similar to other members of the lentivirus family. More importantly, JDV possesses several features in common with HIV that are very attractive as a vector, including the ability to replicate to a high titre (about 10^8 ^plaque forming unit (PFU)/ml of virus in the plasma), and being able to efficiently integrate into chromosomes of non-dividing and terminally differentiated cells. Most of the lentiviral vectors were pseudotyped with a glycoprotein from the vesicular stomatitis virus (VSV), VSV-G, as it provided not only a broad tropism, but also physical strength that enabled concentration by centrifugation.

### (B) Adenoviral vector (AV)

Adenovirus vector is the most commonly studied and most widely used system in cancer gene therapy. They are of particular utility for cancer gene therapy applications, where temporary gene expression is acceptable or even beneficial. The currently employed AVs in clinical trials are all based on serotype 5. AV can be produced at high titre and has demonstrated efficient gene transfer to various types of cancer cells [22]. Two AVs have been approved for clinical use in patients suffering from head and neck cancers in China, one is the adeno-P53 [23], and the other is a replication-competent adenovirus.

Several other adenoviruses, based on canine, porcine, bovine, ovine and avian have all been developed. The ovine AV is based on serotype 7 and developed in Australia. Preclinical testing on prostate cancer in animal models has shown therapeutic efficacy [24].

AVs contain many viral genes encoding major proteins that elicit a strong host immune response. Of particular concern is the development of cytotoxic T lymphocytes that lyse cells expressing the recombinant genes. Newer generations of AV vector were designed to overcome some of these problems and the initial results were encouraging. New techniques involved in removing the recombinant viral genes and transfecting the non-recombinant plasmid with a helper virus and then separating the helper virus with sedimentation techniques were developed. Improvements in helper virus have also been trialled that reduces "floxed" helper virus production 1000-fold, but this method still has a 1% wide type (WT) contamination thus still allowing the possibility of *in vivo *recombination. With regard to AV-mediated cancer treatment, high-level tumour transduction remains a key developmental hurdle. To this end, AV vectors possessing infectivity enhancement and targeting capabilities should be evaluated in the most stringent model systems possible. Advanced AV-based vectors with imaging, targeting and therapeutic capabilities have yet to be fully realized; however, the feasibilities leading to this accomplishment are within close reach [25].

### (C) Adeno-associated virus (AAV)-based vectors

AAV-based vectors have been shown to be non-toxic and undergo widespread cellular uptake in preclinical evaluation [26]. A recent study has compared five different AAV strains and amongst them, serotype 2 was proved to be the most efficient killers of tumour cells. In another study, serotype 8 AAV vector encoding a soluble vascular endothelial growth factor (VEGF) receptor was able to halt tumour growth in several rodent glioma models. However, difficulties in the development of packaging cell lines for AAV, as well as bulk production and vector purification have been reported as problematic [27]. A new system was developed recently to scale up and bulk produce of AAV from insect cells may solve some of these existing problems [27].

### (D) Herpesvirus-based vectors

Vectors based on herpesviruses are well-developed and have progressed to clinical trials. As with other viral vector, replication defective vectors did not show much of use. The first replication competent vector was based on a mutant strain, where the vectors are deleted of the main neurovirulence gene r34.5, which restricted its ability to replicate in adult central nervous system and to form latency. However, later study showed that the mutant strain that had the deletion of the r34.5 gene also reduced the capacity of replication inside tumour cells [28]. The new vector has a deleted ICP47 gene instead without impacting on efficient replication.

Pre-existing immunity may pose a problem that limits the clinical efficacy of herpesvirus-based vectors. The immunity prevented the transduction of peripheral organs and also caused liver toxicity. However, a recent mutant strain-secreting cytokine granule macrophage colony stimulatory factor (GM-CSF) or IL-12 was shown to be effective in liver cancer therapy in a murine model which likely involves both direct viral oncolysis and actions of specific immune effector cells [29].

### (E) Viral replicons and transposons

Semliki Forest virus (SFV) subgenomic replicons (i.e. non toxic replication) have been developed that allow stable expression of a required gene e.g. beta-galactosidase (beta-Gal) in mammalian cell lines. Expression remained high (approximately 150 pg per cell) throughout cell passages [30].

Since construction of the Sleeping Beauty transposon from defective copies of a Tc1/*mariner *fish element [31], new vertebrate genetic manipulation tools (i.e. transposase enzymes) have become available for gene therapy. This particular transposase in the system binds to the inverted repeats of salmonid transposons that surround the insertion gene and mediate precise 'cut and paste' into fish, mouse and human chromosomes. Potential problems with the use of transposons for gene therapy may arise from having no 'off' switch for the transposase and the relatively low quantities of integrated product, either of which would make retroviral intergrase as a more suitable or alternative enzyme for chromosomal integration.

### (F) Targeted viral vectors

While efforts have been focused on the continuing refinement of various vector systems, several obstacles remain, primarily the low efficiency of gene delivery into target tumour cells. The vascular endothelial wall is a significant physical barrier prohibiting access of systemically administered vectors to the tumour cell. To overcome this obstacle, strategies are currently being developed to take advantage of transcytosis pathways through the endothelium. An AV vector targeted to the transcytosing transferrin receptor pathway, using the bifunctional adapter molecule had been constructed [32]. The transcytosed AV virions retained the ability to infect cells, establishing the feasibility of this approach. However, efficiency of AV trafficking via this pathway is poor. Other efforts are directed towards exploring other transcytosing pathways such as the melanotransferrin pathway, the poly-IgA receptor pathway, or caveolae-mediated transcytosis pathways. There are hopes to develop mosaic AV vectors incorporating both targeting ligands directed to such transcytosis pathways as well as ligands mediating subsequent targeting and infection of tumour cells present beyond the vascular wall [33].

### (G) Viral vector-associated multifunctional particles (MFPs)

Recently, a concept of multifunctional particles (MFPs) based viral vectors has emerged. The idea incorporated viral vectors' tumour targeting, imaging and amplifying tumour killing capacities. AAV has been developed as MFP, by virtue of genetic capsid modifications, to incorporate additional functionalities, such as modified fibres, combined with imaging motifs on the pIX protein, to simultaneously target tumour cells while monitoring viral replication and spread. HSV TK has been incorporated at pIX site of the AAV capsid. This enzyme is compatible with available PET imaging ligands such as ^18^F-penciclovir, providing an imaging system for viral replication that can directly be translated for clinical applications. Interestingly, HSV TK is an enzyme that has utility in so-called suicide gene therapy, in which the expressed enzyme converts a substrate such as ganciclovir to its phosphorylated metabolite, which can then be further phosphorylated by cellular kinases to a toxic metabolite, causing cell death [34]. Also, tumour cells expressing this gene product induce the death of adjacent cells via the so-called 'bystander effect', thus representing an 'amplifying strategy' as mentioned above.

Nanotechnology has also been introduced recently in the context of MFP. This is defined as the development of devices of 100 nm or smaller, having unique properties due to their scale. The devices that are being developed generally incorporate inorganic or biological material. In this regard, the coupling of inorganic nano-scale materials to targeted AV vectors has much potential. For example, magnetic nano-particles have recently received much attention due to their potential application in clinical cancer treatment; targeted drug delivery and magnetic resonance imaging (MRI) contrast agents [35]. However, despite the useful functionalities that might derive from metal nanoparticle systems, the lack of targeting strategies has limited their application to locoregional disease. Thus, tumour-selective delivery is the key to improve therapeutic applications of this technology.

## Mechanisms of viral vector-mediated cancer gene therapy

### (A) Corrective gene addition

The p53 tumour suppressor gene has received a great deal of attention as a cancer therapeutic strategy due to the important role it plays in maintaining the integrity of the genome. It is involved in cell cycle regulation, DNA repair, and apoptosis, essentially controlling the integrity of the genome. Following exposure to DNA damaging agents, p53 activation results in cell cycle arrest, allowing for DNA repair or triggers cellular apoptosis if the damage is irreparable. Thus, p53 mutations in cancer allows for unregulated cellular proliferation in the face of genetic mutations and accumulation of genetic errors contributing to the malignant phenotype and genomic instability of cancer cells. Preclinical studies have demonstrated that replacement of *wt-p53 *in cancer cells through gene transfer techniques restores *p53 *function and triggers apoptosis leading to tumour cell destruction [36,37]. The effect is selective to tumour cells with dysfunctional P53 as apoptosis is not triggered in normal cells containing *wt-p53 *following gene transfer.

Based on these preclinical studies a number of *p53 *gene repair clinical trials have been initiated. These trials have used different vector systems for gene transfer (retrovirus and adenovirus), different routes of vector delivery (intra-tumoural injection and bronchial lavage), and have focused on different subtypes of lung cancer (non small cell lung cancer, NSCLC and bronchioloalveolar carcinoma). The first phase I clinical trial of such an approach was conducted by Roth et al. at the M.D. Anderson Cancer Centre [38]. In this trial, nine patients with advanced NSCLC received intra-tumoural injections of a retroviral vector containing *p53 *via CT-guided or endobronchial injections. Effective gene transfer was demonstrated in biopsied tumours following injection and some degree of tumour regression of the injected lesion was seen in three subjects providing proof-of-concept for this gene therapy approach. All subsequent trials have utilized adenoviral vectors for gene transfer since such vectors are relatively easy to manufacture at large scale, can be produced at higher viral titres, and have the ability to transduce both dividing and non-dividing cells. Three adenoviral *p53 *(Ad-p53) single agent trials have been performed as well as three Ad-p53 combination trials. Two of the single agent trials were performed in advanced NSCLC using either single or multiple vector injections [39]. These trials demonstrated minimal toxicity, successful p53 gene transfer, and transient injected lesion tumour regressions. However, a similar proof-of-concept trial utilizing endobronchial injections of an adenoviral vector containing the marker gene, β-galactosidase, also showed localized antitumour responses suggesting that the vector backbone by itself might have antitumour activity regardless of the transgene delivered [40]. Nevertheless, an important observation was that effective gene transfer with minimal toxicity could be achieved with repeated administration even in the face of high-titre neutralizing anti-adenovirus antibodies. Unfortunately no tumour regressions were observed in non-injected lesions providing no evidence for a clinically relevant systemic "bystander" killing effect. Thus, the principal disadvantage of this treatment approach is the theoretical need to genetically modify every cancer cell in a tumour mass to achieve a maximal anti-tumour effect. There were also reported trials of Ad-p53 combined with chemotherapy [41], or radiation. However, no obvious responses to the therapeutic were observed. Early trials utilized *p53 *gene transfer as the sole treatment modality whereas more recent trials have combined *p53 *gene transfer with other cancer therapies, notably chemotherapy or radiation, as part of a combined modality treatment approach.

### (B) Suicide-gene therapy

This is also one of the well studied strategies and is based on the delivery of a "suicide-toxin producing enzyme" gene not normally found in mammalian cells to tumour cells that allows for selective sensitivity to a systemically administered pro-drug. One such suicide gene is the HSV TK gene that codes for an enzyme that converts the normally nontoxic nucleoside analogue, ganciclovir, in subsequent steps into a toxic compound that leads to tumour cell death. Like the adenoviral *p53 *gene transfer approaches described above, preclinical data with adenoviral HSV TK gene transfer (AdHSV TK) followed by ganciclovir exposure results in a bystander effect in which neighbouring, non-transduced cells are also being killed, presumably due to transfer of toxic metabolites from the transduced cells as well as induction of a generalized immune response [42]. Preclinical studies in an immunocompetent, orthotopic lung cancer model demonstrated prolonged survival of mice inoculated with AdHSV TK transfected tumour cells following treatment with ganciclovir compared with controls. While clinical data on this approach has not been reported to date in lung cancer two clinical trials utilizing an adenoviral vector to deliver the HSV TK gene to patients with mesothelioma via intra-pleural administration have been reported [43]. Gene transfer was confirmed in more than half of the patients and several partial tumour regressions were noted. Concomitant administration of systemic corticosteroids in an attempt to suppress the anti-adenoviral immune response in one of the two studies was ineffective, but did demonstrate a trend toward increased gene transfer.

At present, the hurdle to clinical use of this strategy is the low efficiency of gene transfer. To overcome the problem, we also developed a novel way of using a unique peptide to shuttle the HSV TK gene to neighbouring cells [44]. However, clinical efficacy has yet to be shown.

### (C) Immuno-gene therapy

Several genetic strategies have been employed to enhance the immunogenicity of tumours with a goal of inducing immune-mediated tumour destruction. Unlike the gene repair and suicide gene transfer studies described above, immunogene therapies have the theoretical advantage of inducing a systemic anti-tumour response associated with immunologic memory. Such a response potentially allows for treatment of disseminated disease and a prolonged anti-tumour effect that persists beyond the immediate treatment period. Immunogene therapy strategies involve both *ex vivo *and *in vivo *approaches. Early studies of adoptive transfer of *ex vivo *expanded tumour infiltrating lymphocytes (TIL) demonstrated responses in melanoma and renal cell cancer but activity in other solid tumours was limited [45]. Systemic administration of interleukin-2 (IL-2) appeared to enhance the activity of TIL in some trials, but was associated with marked toxicity. In an attempt to enhance the immunologic potency of TIL without the associated toxicities of systemic IL-2 administration, genetic modification of TIL with the gene for IL-2 has been studied. A phase I trial of IL-2 modified autologous TIL in NSCLC has been completed. In this trial TIL were harvested from malignant pleural effusions in patients with NSCLC, genetically modified with an adenoviral vector containing the IL-2 gene, and reinfused into the pleural cavity. Decreases in pleural effusions as well as a partial tumour regression were noted among ten treated patients. A second approach in preclinical development involves genetic modification of dendritic cells with the gene for interleukin-7 (IL-7). IL-7 stimulates cytotoxic T-lymphocyte responses and down-regulates tumour production of the immunosuppressive growth factor, TGF-β. In murine models, intra-tumoural administration of dendritic cells modified with an adenoviral vector containing IL-7 led to tumour regressions and immunologic memory far superior to that seen with direct intratumoural injection of the AdIL-7 vector.

We have developed an *ex vivo *approach using the lentiviral vector-mediated transfer of the tumour antigen gene into dendritic cells (DCs) cells. Therapeutic effects were demonstrated in up to 85% of the subjects [46].

### (D) Anti-angiogenesis gene therapy

One of the features of the malignant tumours was the increased vasculature. Therefore, targeting tumour vasculature rather than the tumour cell itself as a treatment for cancer has gained increasing interest in recent years. A number of inhibitors of angiogenesis (e.g. angiostatin [47], endostatin [48]) have been identified and have been shown to induce tumour regressions in preclinical models through inhibition of tumour neovascularization. An alternative strategy to inhibit tumour angiogenesis is the genetic delivery of genes with anti-angiogenenic properties directly to the tumour vasculature. One of the most promising strategies in preclinical development involves delivery of a mutant *Raf *gene to angiogenic blood vessels using αvβ3-targeted liposomes. The integrin, αvβ3, is preferentially expressed in the angiogenic endothelium and contributes to viral internalization making it a good targeting molecule for anti-angiogenic gene therapy strategies. *Raf *is a cellular signalling molecule that plays an important role in neovascularization. Mice lacking *Raf *die early in development with vascular defects and a mutant form of *Raf *was shown to block angiogenesis in response to pro-angiogeneic factors *in vitro*. Systemic injection of targeted liposomes conjugated to a mutant *Raf *gene into mice with pre-established lung and liver metastases from colon carcinoma demonstrated predominant tumour endothelial cell uptake, tumour endothelial cell apoptosis, and pronounced tumour regressions.

An alternative gene therapy strategy targeting the tumour vasculature is a tumour vaccine targeting the vascular endothelial growth factor receptor-2 (VEGF2, also known as FLK-1). VEGFR2 has relatively restricted expression on endothelial cells and is upregulated in proliferating tumour neovasculature. An orally available DNA vaccine encoding murine FLK-1 was shown to suppress angiogenesis in tumour vasculature, protect mice from lethal challenges with melanoma, colon, and lung carcinoma cells, and reduce the growth of established metastases [49].

### (E) Gene silencing

One of the newer technologies in cancer gene therapy involves the silencing of genes in cancer cells that regulate tumour cell growth and proliferation. We have developed a double stranded RNA mediated silencing of the epidermal growth factor receptor (EGFR). In vitro studies have demonstrated effective silencing of the EGFR and resulted *in the *growth inhibition of NSCLC cells. *A f*urther study is underway to demonstrate the in vivo efficacy of EGFR silencing in animal models [50]"

## Clostridial spores specifically target and deliver therapeutic genes to tumours

It is obvious that a major step towards the development of an effective cancer therapy will be to construct a vector that targets the tumour alone, and is capable of spreading to and throughout the tumour found in tissues. Clostridial spores fit into this equation very well. Clostridia are strictly anaerobic. They are gram-positive, rod-shaped, and form spores under unfavourable conditions. There are about 80 species and several of these have been tested in solid tumours. All known species require anaerobic conditions to grow but do vary in their oxygen tolerance and their biochemical profile. Clostridial spores have been administered intravenously and showed a distinct advantage for use in cancer therapy as they are easy to produce and store. Germination of spores will only occur when they encounter the requisite anaerobic conditions. Spontaneous colonization of tumours in cancer patients and the apparent selectivity of Clostridia for tumours were noticed more than 50 years ago. The first experiment in 1947 showed that direct injection of spores of *C. histolyticum *into mouse sarcoma caused oncolysis (liquefaction) and tumour regression [51]. Later experiments proved this selectivity by injecting mice *i.v*. with spores of C. tetani, the causative agent of tetanus. Injected non-tumour bearing animals remained healthy. However, tumour bearing mice died within 48 h because of *C. tetani *colonisation and tetanus production. This provided evidence that the *C. tetani *were able to germinate/replicate selectively in the anaerobic environment found within tumours, and released their toxins systemically [52]. Obviously, it would not be appropriate to use pathogenic strains of Clostridia for clinical therapy in humans. A non-pathogenic strain of *C. butyricum *M-55 has been isolated [53]. M-55 was later reclassified as *C. oncolyticum *and taxonomic studies have now clearly established that it is a *C. sporogenes *strain (ATCC13732). This is a proteolytic species causing liquefaction of colonised tumours. This was later verified by testing more isolates.

Saccharolytic clostridia, such as *C. beijerinckii *NCIMB8052 spores administered intravenously to EMT6 tumour-bearing mice germinated in the necrotic tumour regions while the oxygenated tumour areas remained free of spores [54]. Equally, intravenous injection of rhabdomyosarcoma-bearing rats with at least 107 spores of *C. beijerinckii *ATCC17778, C. acetobutylicum DSM792 (= ATCC824) or *C. acetobutylicum *NI-4082 (reclassified as C. saccharoperbutylacetonicum) showed tumour colonisation without complete tumour lysis [55].

*C. sporogenes *was the first Clostridium to be gene modified and this was performed with the E. coli Colicin E3 gene. Colicin E3 encodes a bacteriocin shown to have canceriostatic properties [56]. However, the overall anti-tumour efficacy of this bacteriocin was limited. This may have resulted from poor gene modification methodologies which were improved with the application of electroporation. In 2002 Prof. Brown's group introduced *E. coli *cytosine deaminase (CD) into *C. sporogenes *NCIMB10696 by electroporation [57]. Intravenous injection of the recombinant spores followed by the systemic administration of the prodrug 5-FC inhibited tumour growth which was more pronounced than the use of prodrug alone. Unfortunately, for reasons unknown this inhibition in tumour growth did not persist. However, it was clear that *C. sporogenes *has a great capacity to colonise the tumour. At least 10e8 CFU/g of tumour was obtained following the intravenous injection of the spores (Table 1).

**Table 1:** Genetically modified recombinant clostridial strains and their antitumour studies.

Recombinant Strain	Model	Strategy/Result	Reference
*C. oncolyticum/sporogenes*recombinant for *E. coli *colicin E3	*In vitro *study	Cancerostatic properties	[56]
*C. beijerinckii *(*acetobutylicum*) recombinant for *E. coli *cytosine deaminase (CDase)	*In vitro *study and tested on murine EMT6 carcinoma cell-line	Sensitivity to 5-fluorocytosine increased 500-fold	[72]
*C. beijerinckii *(*acetobutylicum*) recombinant for Nitroreductase (NTR)	EMT6 Mouse Prodrug: CB1954	CDEPT strategy with CB1954 Nitroreductase activity detected in tumor lysate	[54]
*C. acetobutylicum *recombinant for Tumour necrosis factor (TNF-α)	Rhabdomyosarcoma	Recombinant protein detected in tumour, but no control of tumour growth	[58]
C. *acetobutylicum *recombinant for *E. coli *cytosine deaminase (CDase)	Rhabdomyosarcoma Prodrug: 5-FC	CDEPT strategy Cytosine deaminase activity detected in tumor lysate	[64]
*C. sporogenes *recombinant for cytosine deaminase (CDase)	SCCVII tumours into syngeneic C3H/Km mice Prodrug 5-FC	Growth delay of tumours	[57]
C. *acetobutylicum *recombinant for interleukin-2 (IL-2)	Rhabdomyosarcoma	Enhanced antitumour effect	[60
*C. sporogenes *and *C. novyi-NT *recombinant for Nitroreductase (NTR)	Human colorectal carcinoma (HCT116)	CDEPT strategy with CB1954 High level of colonization 10^8^–10^9 ^cfu/g tumour. Repeated CDEPT treatment cycle, significant tumour growth delay	[61]

Description of additional data files (N/A)

Saccharolytic Clostridia strains including *C. beijerinckii *ATCC17778, *C. acetobutylicum *DSM792 (ATCC824) or *C. acetobutylicum *NI4082 (reclassified as *C. saccharoperbutylacetonicum*) and *C. butyricum *are non-pathogenic and their development has been industry funded. Therapeutic genes, encoding the cytokine tumour necrosis factor *alpha *(TNF-α), CD or nitroreductase (NTR) have been introduced into these strains [58,59]. Following transformation of *C. acetobutylicum *using strain-specific electroporation protocols, CD expression was monitored in lysates and supernatants of early logarithmic growth phase cultures of recombinant *C. acetobutylicum *(pKNT19closcodA) [12]. A considerable amount of heterologous protein was expressed and efficiently secreted. Also, *C. acetobutylicum *strains NI4082 and DSM792 engineered to produce cytosine deaminase were able to express and secrete this enzyme at the tumour site [58,59]. Functional CD enzyme was detected in the tumour of rhabdomyosarcoma-bearing WAG/Rij rats that were injected with the recombinant *C. acetobutylicum*, but not in control animals. Animals, concomitantly treated with antivascular chemical agent, CombreAp, showed higher incidence of CD-positive tumours (100 versus 58%). Moreover, the level of active CD in these tumour specimens was considerably higher (mean conversion efficiency of 5-FC to 5-FU ~11%) as compared to tumours not treated with the vascular targeting drug (mean conversion efficiency of 5-FC to 5-FU ~11%) when compared to untreated tumours (mean conversion efficiency of 5-FC to 5-FU ~3%) [59]. However, when these recombinant strains were used in solid tumour models *in vivo*, there was a consistent lack of significant tumour regression observed. Factors that may have contributed to this lack of efficacy include a low level of bacterial colonisation of the tumour or insufficient recombinant gene expression and secretion at the tumour site [60]. Recent studies have reported the development of vectors utilising super tumour coloniser *Clostridial *strains *C. sporogenes *or *C. novyi-NT*. Recombinant *C. sporogenes *and *C. novyi-NT *overexpressing NTR showed significant *in vivo *anti-tumour effects [61] when used with prodrug demonstrating the clinical potential of these vectors (Table 1).

## Advantages of clostridial spores as "trojan horse" vectors for cancer gene therapy

At present, there are various gene therapy vector systems under development against cancer. However, due to the complexity of the solid tumours involving angiogenesis, hypoxia, stromal cell, tumour cell heterogeneity and the emergence of de-differentiated stem cells, none of the existing vectors are holding any real promises. The clostridial spore-based vector system is not infectious, and has gained renewed interest, because of the following true advantages.

### (1) Safety

Safety is always a concern when live vector systems are used for human gene therapy. Some of the hurdles of using viral vectors include: (1) whether the vector is sufficiently targeted to tumour alone; (2) whether the vector expresses low levels of viral genes that may lead to increased toxicity and immunogenicity [62]; (3) possible immunogenicity of the transgene that may be reduced with a reduction in the duration of gene expression [63]; and (4) whether viral particles are sequested within the target cells or secreted into body fluid such as urine and subsequently spread into environment. We postulate that the use of clostridial spore based vectors may be a safer option to using viral vectors. Clostridia are strictly anaerobic, are tumour targeted and would be unable to live in non-hypoxic environments. A recent experiment with *C. novyi-NT *has demonstrated that the strain was unable to colonise artificially created infarcted heart where the level of hypoxia was inadequate to support the replication of the Clostridia. Early trials of non-pathogenic Clostridia strains in patients have demonstrated safety. In the unlikely event of an adverse effect, clostridia can be eliminated from the blood stream with the use of readily available antibiotics such as metronidazole which showed total spore clearance from the blood stream after 9 days of treatment [64].

### (2) "Thriving on" the unique tumour microenvironment

The biological properties of virus-based vectors, in particular the ability to enter and replicate (in the case of replication-competent viral vectors) within a tumour cell and then spread from cell to cell are highly relevant for effective cancer therapy. However, recent understanding of tumour pathology has revealed that several features of the tumour environment may not be conducive for viral replication [65,66]. Hypoxia is an important feature of solid tumours and the ability of viruses to enter and replicate in hypoxic cells may be a critical determinant for the success or failure of viral vector-mediated cancer gene therapy. Turning off protein translation is a central process in the cellular adaptation to many types of stress, including viral infection and hypoxia. The hypoxic cells, the apoptotic cells, the quiescent cells are all refractory to viral entry and replication [67]. This is a major problem for virus-based vectors because if the vector can't reach a tumour cell, it can't act or deliver a therapeutic gene. On the contrary, clostridial spores are able to home in on these niche environments because of their own unique metabolic need, which enable them to utilise the tumour micro milieu and respective tissues for their own proliferation. Both wild-type and genetically modified Clostridia have been demonstrated to specifically colonise and destroy solid tumours. "Trojan horse" vectors have further created improved features that enable them to kill tumour cells through multimodality mechanisms.

### (3) Easy production

All of the viral vector systems need sophisticated cell culture systems, expensive culture media, rounds of filtrations and purifications and dedicated centrifugation and storages. On the contrary, clostridial spores can be easily and inexpensively produced from anaerobic bacterial culture. There are only a few steps involved and the spores, once produced can be stored at room temperature for at least 3–6 months.

### (4) Easy administration

While most viral vectors have to be intratumourally injected, intravenous injection of resuspended clostridial spores are possible and sufficient as they will be leaked out of the incomplete vessels in the solid tumour, thus specifically targeting to and colonising the hypoxic regions of the tumours.

### (5) Destruction of all types of cells in the tumour, including stromal cells and stem cells

Solid tumours comprise not only malignant cells, but also extracellular matrix and many other non-malignant cell types, including stromal cells such as fibroblasts, endothelial cells and inflammatory cells. The mechanisms of clostridial vector-mediated tumour killing consist of several aspects: one is from its transgene that encodes prodrug converting enzymes for suicide-gene therapy or cytokines for immuno-gene therapy. These are essentially the same as the viral vectors. However, there is another side of the tumour killing effect that is resulting from the consequences of an innate antitumour effect of the clostridial strain due to production of hydrolytic enzymes including proteases, lipases, and nuclease. Furthermore, there is also a nutrients competition between the clostridia and cells surrounding them (including tumour cells, stromal cells and stem cells), where the clostridia multiplied much faster than the mammalian cells. The cumulative multiplications and the combined events of energy and substance metabolism effectively depleted the limited nutrient source and deprive the tumour cells, causing starvation and death. More recently, there were observations that indicate the germination of the clostridial spores, the transformation from spores to vegetative rods, and the continue multiplications of the vegetative rods inside the tumour activated the immune system, assisting the antitumour effects [68]. These tumour killing mechanisms destroy not only tumour cells, but also any other cells in their vicinity. These are characteristics that viral vectors are not so well equipped, nor any existing convectional cancer therapies.

### (6) Extracellular agent, no cell entry, no gene integration and no mutagenesis

While viral vectors need access to viable target cells and their cellular machinery to achieve transgene delivery and expression, this goal is often difficult to fulfil as some tumour cells are not viable at the time of gene delivery. Furthermore, none of the existing vector systems efficiently transfer genes to every tumour cell which subsequently allows for tumour regrowth. On the other hand clostridial spore replication is not tumour cell dependent and occurs via rod multiplication extra-cellularlly. Furthermore, the tumour killing mechanism of clostridial spores may operate independently of the requirement for gene transfer. Without the requirement for gene integration into the host cell genome removes the possibility of insertional mutagenesis when using Clostridia. Therefore, Clostridia may show tumour killing irrespective of the tumour cell heterogeneity found within the tumour environment.

### (7) No limit on accommodating therapeutic genes

One of the primary limitations of most viral vectors has been the small size of the virion, which only permits the packaging of very limited sizes (usually a few kilobases) of exogenous DNA that includes the promoter, the polyadenylation signal and any other enhancer elements that might be desired. However, for clostridia size limitations are far less restricted, not only because the plasmids used can harbour much larger DNA fragments, but in case the foreign gene is integrated in the host chromosome there is in fact unlimited capacity for insertion of therapeutic genes, forecasting the promising future for the development of ever powerful vectors.

## Conclusion

The unique pathophysiology of solid tumours presents a huge problem for the conventional therapies. Thus, the outcomes of current therapies are so far disappointing. Several new approaches aiming at developing effective treatments are on the horizon. These include a variety of virus-based therapy systems [69-71]. Amongst all these, replication-competent viral vector-mediated cancer therapy is most promising [2,3]. However, even this system suffers from several deficiencies: First, the vectors currently have to be injected intratumourally to elicit an effect. This is far from ideal as many tumours are inaccessible and spread to other areas of the body making them difficult to detect and treat. Second, because of the heterogeneity within a tumour, the vector does not efficiently enter and spread to sufficient numbers of tumour cells. Third, hypoxia, a prevalent characteristic feature of most solid tumours, reduces the ability of the viral vector to function and decrease viral gene expression and production. Consequently, a proportion of the tumour mass is left unaffected and capable of re-growing. Fourth, pre-existing immunity pose a problem for the efficacy of viral vectors. Therefore, there have rarely been any cures with the use of the system.

The strictly anaerobic clostridia, on the other hand, have been shown to selectively colonise in solid tumours when delivered systemically and has resulted in high percentage of "cures" of experimental tumours. A phase I clinical trial combining spores of a non toxic strain (*C. novyi-NT*) with an antimicrotubuli agent has been initiated [10]. Genetic manipulation of clostridia to make them into "Trojan horse" vectors will provide further tumour killing mechanisms and amplifying antitumour effects. Clearly, it is just a matter of time that a "Trojan horse" type of clostridium will become a clinical reality, especially if we can further improve upon the system by providing additional features, ideally including (i) targeting tumours only and not anywhere else, (ii) able to effective kill primary tumours as well as metastases. Current technologies are in place to achieve these goals. Newer and effective therapies for solid tumours based on the "Trojan horse" will be a reality in a very near future.

## Abbreviations

Adenoviral vector (AV); Adeno-Associated Virus (AAV); *C*. clostridium; Cytosine deaminase (CD); Dendritic cells (DCs); Colony forming unit (CFU); Plaque forming unit (PFU); Epidermal growth factor receptor (EGFR); Herpes simplex thymidine kinase (HSV TK); Interleukin-2 (IL-2); Granule macrophage colony stimulatory factor (GM-CSF); Human Immunodeficiency Virus (HIV); Jembrana Disease virus (JDV); Magnetic resonance imaging (MRI); Murine Moloney leukaemia virus (MoMLV); β-galactosidase (β-β-Gal); Multifunctional particles (MFPs); Nitroreductaes (NTR); Non small cell lung cancer (NSCLC); Semliki Forest virus (SFV); Severe combined immunodeficiency syndrome (SCIDs); tumour infiltrating lymphocytes (TIL); Tumour necrosis factor (TNF); Vascular endothelial growth factor (VEGF); Vesicular stomatitis virus (VSV); Wide type (WT).

## Competing interests

The author(s) declare that they have no competing interests.

## Authors' contributions

All authors participated in the production of the manuscript together and have read and approved the final manuscript.
